# Sustainable Extraction and Structural Characterization of Cellulose from Spent Coffee Grounds via Combined Ultrasound and Alkali–Urea Pretreatment

**DOI:** 10.3390/polym18151893

**Published:** 2026-08-01

**Authors:** Mae Salman, Lilia Tafran, Idrisa Kiryowa, Pankaj Kumar, Fares Dia Eddine Ghorabe, Raed Alayouni, Tamer M. El-Messery, Mohamed Said Boulkrane

**Affiliations:** 1 Faculty of Biotechnologies, ITMO University, Lomonosova St. 9, St. Petersburg 191002, Russia; 408066@niuitmo.ru (M.S.); lilyatafran@gmail.com (L.T.);; 2 Infochemistry Scientific Centre, ITMO University, Lomonosova St. 9, St. Petersburg 191002, Russia; 3 Department of Food Science and Human Nutrition, College of Agriculture and Food, Qassim University, Buraydah 51452, Saudi Arabia; r.alayouni@qu.edu.sa

**Keywords:** spent coffee grounds, sustainable, cellulose extraction, ultrasound-assisted, urea, valorization, polymer

## Abstract

The combined pretreatment of ultrasound irradiation, urea, and sodium hydroxide was explored to increase the extraction and delignification of cellulose from spent coffee grounds (SCGs), shortening the extraction time. The impacts of different experimental conditions, namely alkali concentration (5 vs. 10%), urea addition (0 vs. 3%), and ultrasound aid (30 min, 40 kHz, 150 W), on the physicochemical characteristics of the extracted cellulose were determined. The first characteristic of the raw biomass was its high lignin content (30.4%), necessitating a rigorous delignification process. To confirm this, samples were further analyzed by FTIR, XRD, TGA, and SEM. The successful elimination of non-cellulosic material, as indicated by the disappearance of the carbonyl stretch at 1730 cm^−1^ and attributed to hemicellulose and pectin, was confirmed by FTIR. The XRD results revealed that while the individual application of ultrasound yielded the highest apparent crystallinity index (64.5% for sample B), the synergistic low-alkali combination with urea (sample F) resulted in a lower, controlled crystallinity index (56.2%). This indicated that the coexistence of urea and ultrasound induces significant fiber swelling and localized hydrogen-bond disruption, as well as highly efficient chemical purification. The thermal analysis revealed that all samples had high thermal stability with a maximum degradation temperature (T_max_) between 316 and 322 °C, which can be used in composites. The process was visually confirmed by morphological analysis carried out under SEM, which revealed a progressive surface erosion of the compact lignocellulosic matrix into cellulose-rich samples treated with ultrasound.

## 1. Introduction

The coffee industry generates significant bio-waste [1], with an estimated 6–10 million tons of spent coffee grounds (SCGs) [2] produced annually as a byproduct of soluble coffee manufacturing [3,4,5]. Currently, these residues are largely disposed of in landfills or incinerated, leading to methane emissions and the release of organic pollutants, such as caffeine and tannins, into the environment [6]. However, SCGs are a highly underutilized renewable resource, chemically comprising 45–50% polysaccharides (cellulose and hemicellulose) along with lignin and proteins [7,8]. Valorizing these structural polymers into high-value bioproducts aligns with circular economy principles, transforming an environmental liability into a sustainable green feedstock [3]. Although traditional methods for extracting cellulose from lignocellulosic biomass typically involve strong alkaline solutions as a pretreatment to weaken lignin–carbohydrate complexes and solubilize hemicellulose, such as NaOH concentrations over 8% (*w*/*v*) [9] and often combined with elevated temperatures (>100 °C), they still present several sustainability issues, including high energy consumption, excessive chemical use, and alkaline waste. Such disadvantages reduce scalability and environmental compatibility, particularly in small- and medium-sized businesses that have the desire to valorize SCG residues at the local level. Recent reports have highlighted the potential of ultrasound-assisted processes to enhance mass transfer and delignification of biomass through pretreatments [10]. Ultrasonic cavitation produces microjets and shockwaves that disrupt cell walls, enhancing solvent penetration and increasing cellulose availability [11]. Urea, conversely, has been noted as a low-cost hydrogen-bonding agent that can be used to swell cellulose fibers by destabilizing intermolecular hydrogen bonds, allowing them to loosen and increase their solubility in solvents [12]. Although these are individual achievements made in the extraction of polymers, little attention has been given to the synergistic process of using ultrasound and alkali–urea mechanisms in cellulose extraction, as well as that of SCGs. Similar synergistic actions with other lignocellulosic feeds are examined in a couple of related studies, e.g., Miscanthus × giganteus [10], where pretreatment with ultrasound-assisted alkali–urea improved cellulose yield and reduced NaOH concentration significantly. Nevertheless, these combined strategies have not been optimized systematically in the context of SCGs, which possess unique structural properties, such as increased lipid levels and melanoidin-bound carbohydrates, which might impede the penetration and extraction efficacy of the solvent [6]. Deep eutectic solvents (DESs) have been combined with urea to extract cellulose from agri-food residues, although their application is limited to SCGs [9]. The existing gap in understanding how ultrasonic cavitation and urea-mediated fiber swelling can work together to improve the efficiency of cellulose extraction from spent coffee grounds (SCGs) under milder chemical and thermal conditions provides a solid foundation for significantly reducing chemical use and environmental impact while also increasing the economic viability of SCG valorization methods. This paper hypothesizes that the synergistic effects of ultrasonic cavitation and urea-mediated swelling can be highly utilized to stimulate the mass transfer of lignocellulosic matrices and the destruction of spent coffee grounds, as this approach will decrease the concentration of NaOH without decreasing the yield or purity of cellulose. This process is enhanced by the addition of ultrasound energy to an alkali–urea solvent system to create a low-impact route to extract cellulose from SCGs. This strategy will not only lead to sustainable waste valorization but also facilitate the development of green processing technologies in line with the objectives of the circular bioeconomy.

## 2. Materials and Methods

### 2.1. Materials and Reagents

The used coffee grounds were a blend of Coffea arabica and Coffea canephora (Robusta) and were provided from coffee shops in Saint Petersburg, Russia. Sodium hydroxide (NaOH), urea, hydrogen peroxide (H_2_O_2_), sulfuric acid (H_2_SO_4_), n-hexane, acetone, ethanol, and distilled water were procured from Chem Express (Russia), and all the chemicals were of analytical grade.

### 2.2. Methods

#### 2.2.1. Raw Material Preparation

The SCGs were collected and immediately treated according to the procedure by Oliva [13] and the samples were then frozen at −18 °C to prevent microbial growth and enzymatic activity [13,14]. All the collected samples were defrosted, dried at 60 °C in an air oven for 24 h to achieve a uniform mass, and then ground in a laboratory grinder (<500 μm) to enhance sample uniformity and the efficiency of subsequent mass transfer [7]. These were stored in sealed containers for further processing. The samples’ moisture content was assessed using the standard gravimetric oven method [15]. Samples were desiccated at 105 ± 5 °C for 24 h or until a consistent weight was attained. Following pre-drying at 60 °C, the moisture content of the prepared SCG samples was 5.80 ± 0.6%. The samples’ ash concentration was 1.94 ± 0.3% (*w*/*w*), which was ascertained by heating the samples in a muffle furnace at 550 °C for 6 h [15].

#### 2.2.2. Defatting

Defatting was a necessary preparation procedure involved in extracting the inherent lipophilic constituents from the spent coffee grounds (SCGs), including coffee oils, coffee waxes, and triglycerides, which can make up to 10–15% of the dry weight [1]. These hydrophobic compounds are likely to cover the lignocellulosic structure, inhibiting the diffusion of reagents and greatly reducing further alkali-based delignification and cellulose separation [11]. To solve this, n-hexane was utilized as a nonpolar solvent in a solid/solvent ratio of 1:5 (*w*/*v*) for Soxhlet extraction, which was carried out under a continuous 6 h reflux cycle [16]. However, while n-hexane was utilized as a standard nonpolar laboratory solvent in this Soxhlet extraction to guarantee a completely oil-free matrix for clear subsequent delignification, its application creates an operational contradiction with the stated small- and medium-sized enterprise (SME) sustainability goals. Hexane is widely recognized as a hazardous neurotoxicant, with high flammability and severe occupational health risks. To maintain alignment with green chemistry principles during industrial scale-up, n-hexane must be substituted with eco-friendly green bio-solvents, such as bio-derived ethanol, supercritical ${\mathrm{CO}}_{2}$, or 2-methyltetrahydrofuran (2-MeTHF).

The process allows for the percolation of solvents through the biomass multiple times, which guarantees the proper dissolution and extraction of oils and other extractives. The defatted material was then dried in the oven at 60 °C until the required constant mass was obtained to remove any remaining solvents. The resulting powder was found to have increased wettability and porosity, thus increasing alkali penetration and overall activity of the cell wall polymers during the pretreatment stage. This pretreatment step does not just standardize the chemical composition of SCGs, it also makes the subsequent cellulose extraction represent the actual efficacy of the applied alkali–urea and ultrasound-assisted systems and not merely that of lipid interference [17].

#### 2.2.3. Weight Loss Determination

The weight loss of the extracted cellulose was determined gravimetrically at each step of the extraction procedure. After oven-drying the samples and ensuring a constant weight, measurements were taken to calculate the mass lost during each stage of the process [18]. To determine the yield percentage, the mass of dried cellulose recovered after extraction is divided by the initial mass of defatted SCGs [19]. The ratio was then converted into a percentage to calculate the efficiency of the process, indicating how much pure cellulose was successfully isolated from the oil-free coffee waste after removing lignin and hemicellulose.

Yield (%) = (Mass of dried cellulose/Mass of defatted SCGs) × 100

#### 2.2.4. Chemical Composition

Raw SCGs have a chemical composition that consists of cellulose, hemicellulose, and acid-insoluble lignin [20]. Sequential chemical fractionation was used to determine the lignin and ash content [9].

Acid-Insoluble Lignin: According to the method of Mussatto et al. [5], 1.0 g of dried SCGs was treated with 10 mL of 72% H_2_SO_4_ at room temperature, diluted to 1 N, and then refluxed at 100 °C for 1 h. The hydrolysate was then filtered using pre-weighed filter paper and neutralized. Oven-drying of the solid residue was done at 105 °C to a constant weight. The percentage of acid-insoluble lignin was calculated according to the following formula:

Lignin (%) = [(W_2_ − W_1_)/W_0_] × 100.

where W_0_ = the initial dry mass, W_1_ = the filter paper mass, and W_2_ = the filter + residue mass.

Ash Content: The residue, including the lignin and filter paper, was burned in a muffle furnace at 550 °C for 3 h. The cooled ash was weighed after letting the contents cool (W_3_). Then, the ash content was calculated using the following formula:

Ash (%) = (W_3_/W_0_) × 100

Cellulose: A 1.0 g sample of dried SCGs (W_4_) was accurately weighed and put in a 250 mL round-bottom flask, and 100 mL of 1 N sodium hydroxide (NaOH) was added to it. The suspension was left at 80 °C with continuous magnetic stirring for 60 min to achieve a homogeneous reaction. In the process, the lignin and hemicellulose were hydrolyzed by NaOH, and the hemicellulose fraction was solubilized in the alkaline solution selectively, while the cellulose and lignin remained insoluble as an insoluble residue [21].

Hemicellulose (solubilized) + NaOH → Soluble alkali hemicelluloses

The suspension was filtered through a vacuum under a pre-weighed filter paper following alkaline extraction. The solid residue was well washed with distilled water until a neutral pH (about 7) of the filtrate was attained to remove the remaining alkali and soluble degradation products. The residue was dried in an oven at 105 °C until reaching a constant weight (W_5_), which was the summative weight of the cellulose and lignin. The content of cellulose was calculated as follows:

Cellulose (%) = [(W_5_ − W_2_ + W_1_)/W_0_] × 100

where W_5_ = the mass of the residue after NaOH extraction (g).

Cellulose Purity (%): The purity of cellulose was determined using a rigorous quantitative mass-balance approach. Following sequential chemical fractionations, the non-cellulose mass fractions remaining in the isolated solids were determined via acid hydrolysis, and the purity was calculated according to the following equation:

$$\mathrm{Cellulose}\;\mathrm{Purity}\;(\%)=\left(1-\frac{W_{\mathrm{residual}\;\mathrm{lignin}}+W_{\mathrm{residual}\;\mathrm{hemicellulose}}}{W_{\mathrm{final}\;\mathrm{isolated}\;\mathrm{sample}}}\right)\times 100$$

where $W_{\mathrm{final}\;\mathrm{isolated}\;\mathrm{sample}}$ represents the total dry mass of the recovered sample, and the impurity fractions are cross-validated by the persistent FTIR peak ratios at 1730 ${\mathrm{cm}}^{-1}$ and $1510\;{\mathrm{cm}}^{-1}$.

Hemicellulose Determination: Hemicellulose was solubilized during the alkali pretreatment; its percentage ratio was determined by a mass-balance method used on the overall make-up, in which the fractions of cellulose, lignin, and ash, as well as any previously determined extractives, were integrated. This process assumes that there are only a few losses in further washing and filtration, which is highly consistent with the general analytical standards of lignocellulosic feedstock [14]. As a result, the content of hemicellulose is calculated using the formula provided below:

Hemicellulose (%) = 100 − (Cellulose (%) + Lignin + Ash + Extractives)

where extractives refers specifically to the lipophilic constituents (coffee oils, waxes, and triglycerides) removed during the initial 6 h Soxhlet extraction with n-hexane, as well as any water-soluble proteins or low-molecular-weight phenolic compounds removed during subsequent washing steps.

#### 2.2.5. Extraction and Bleaching of Cellulose

To facilitate comparison among the different extraction approaches, the pretreatment conditions applied to spent coffee grounds (SCGs) were coded as treatments A–F according to the NaOH concentration, urea addition, and ultrasound application. The treatment combinations used throughout the study are summarized in Table 1.

Following the treatment scheme presented in Table 1, cellulose extraction and bleaching were carried out as described below.

##### NaOH Extraction

Spent coffee grounds were treated with NaOH solutions (5% and 10%, *w*/*v*) in a solid–liquid system under continuous magnetic stirring. The treatment was carried out for 90 min at a controlled temperature (between 60 and 80 °C). The solids were collected after extraction following alkali treatment and then separated before being vacuum-filtered and washed to a neutral pH (about 7.0) with distilled water. After that, they were subjected to a bleaching step using hydrogen peroxide (H_2_O_2_) [10,22]. A ratio of 1:10 (*w*/*v*) solid to liquid was then used with 15% (*w*/*v*) of hydrogen peroxide to bleach the samples for 1 h at 60 °C with gentle stirring. The pH of the bleaching solution was then carefully adjusted to a slightly alkaline solution (pH about 10) to induce oxidative delignification according to Ravindran et al. [23]. Finally, it was allowed to stand overnight to completely oxidize. The bleached substance was then carefully washed with distilled water until the level of neutral pH was obtained and then dried to be further characterized.

##### Ultrasound–NaOH Extraction

The spent coffee grounds were initially subjected to the ultrasound-assisted process, where the grounds were subjected to 30 min of ultrasound treatment (40 kHz, 150 W) in the NaOH solution (5% and 10% NaOH). The ultrasonic treatment was used to stimulate the physical disruption of the biomass structure and increase the penetration of the reagent [10,12,24]. The samples were exposed to identical chemical treatment conditions as mentioned with the NaOH extraction after exposure to the ultrasound process, with subsequent bleaching with hydrogen peroxide for 1 h at 60 °C and overnight standing. The process of subsequent washing to a neutral pH and drying was carried out just like the rest of the treatments [10].

##### NaOH–Urea Extraction

In the NaOH–urea extraction process, the same experiment procedure used for NaOH extraction was used, except that it was supplemented with 3% (*w*/*v*) urea in the alkaline solution. Spent coffee grounds were added to a freshly prepared NaOH–urea solution, and the solution was stirred for 90 min at 60–80 °C. Once the alkali–urea treatment was completed, the same process was repeated with hydrogen peroxide bleaching (1 h at 60 °C) of the samples and then left to stand overnight. Each treatment was directly comparable to the others, as washing and drying were performed in the same way as in the standard NaOH extraction.

##### Ultrasound–NaOH–Urea Extraction

The spent coffee grounds were given 30 min of ultrasound treatment (40 kHz, 150 W) in the solution of NaOH–urea (5% and 10% NaOH with 3% urea) [12]. Ultrasonic treatment was applied to promote physical disruption of the biomass structure and enhance reagent penetration. After ultrasound exposure, the samples were subjected to the same chemical treatment conditions as described for the NaOH–urea extraction, including bleaching with hydrogen peroxide for 1 h at 60 °C and overnight standing. Subsequent washing to a neutral pH and drying were performed identically to the other treatments.

The cellulose yield (%) was determined using the following formula:

Yield (%) = (Mass of dried cellulose/Mass of defatted SCG) × 100

#### 2.2.6. Characterization Techniques

Thermogravimetric Analysis (TGA): The thermal stability and degradation of the extracted cellulose were studied using a NETZSCH TG 209F1 Libra, Selb, Germany). (TGA) from Germany. The analysis involved heating a sample, about 5–10 mg, from 25 °C to 900 °C. This was done at a rate of 10 °C per minute. The heating process was done in a nitrogen atmosphere, which was kept free of other gases by using a flow rate of 50 mL/min.

X-Ray Diffraction (XRD): XRD was utilized to identify the crystallinity and phase structure of the extracted cellulose. The crystallinity of the samples was assessed using a Bruker X-ray diffractometer (Bruker D2 PHASER, Karlsruhe, Germany). The diffractometer was operated at a voltage of 30 kV and a current of 30 mA using Cu Kα radiation (λ = 1.5406 Å). An initial scan of the first sample (sample A) was performed over a 2θ range, from 10° to 50° with a step size of 0.02, to determine the complete diffraction profile. Since no additional characteristic cellulose diffraction peaks were detected beyond 35°, the remaining samples (from B to F) were scanned over a 2θ range, from 10° to 35° with a step size of 0.02°. The resulting diffraction patterns were analyzed using the DIFFRAC.SUITE to determine the crystalline and amorphous phases and verify the results in OriginPro8.

Fourier Transform Infrared (FTIR) Spectroscopy: The FTIR spectroscopy method was applied to evaluate vibrations of the molecular capacities of particular functional groups of the extracted cellulose. The spectra were observed at wavelengths between 4000 and 500 cm^−1^. Ground samples were scanned by an FTIR spectrophotometer (IRAffinity-1, Shimadzu, Kyoto, Japan) equipped with an attenuated total reflection accessory. Each spectrum was acquired by averaging 32 scans per sample.

Scanning Electron Microscopy (SEM): A Czech Republic electron microscope, TESCAN Vega 3, was utilized to morphologically characterize cellulose fibers and view the topography of the surfaces and structural changes caused by pretreatments. Samples were attached to the aluminum stubs using carbon adhesive tape and sputtered with a thin layer of gold (10 nm) to avoid charging during the imaging process. Micrographs were obtained at an accelerating voltage of 5 kV.

#### 2.2.7. E-Factor Calculations

To quantitatively evaluate and validate the process sustainability claims beyond qualitative statements, the Environmental Factor (E-factor) was calculated according to standard green chemistry parameters:

$$E\text{-}\mathrm{factor}=\frac{\mathrm{Mass}\;\mathrm{of}\;\mathrm{Total}\;\mathrm{Waste}\;\mathrm{Generated}\;(\mathrm{kg})}{\mathrm{Mass}\;\mathrm{of}\;\mathrm{Final}\;\mathrm{Isolated}\;\mathrm{Product}\;(\mathrm{kg})}$$

For the designated optimal low-alkali process (sample F), starting with an initial dry mass of 20 g 0.020 kg of raw SCGs and obtaining a final dry cellulose mass of 5.201 g (0.005201 kg), the organic mass-only E-factor (omitting standard recyclable washing solvents and water) can be calculated as:

$$E\text{-}\mathrm{factor}=\frac{0.020\;\mathrm{kg}-0.005201\;\mathrm{kg}}{0.005201\;\mathrm{kg}}=\frac{0.014799\;\mathrm{kg}}{0.005201\;\mathrm{kg}}\approx 2.85$$

An E-factor of 2.85 demonstrates that approximately 2.85 kg of non-cellulosic byproduct organic waste (lignin, hemicellulose, and natural oils) is generated per 1 kg of purified cellulose product. This matches the fine chemical manufacturing benchmarks and visually validates the low environmental impact of the process.

#### 2.2.8. Statistical Analysis

All experimental procedures, including chemical composition profiling, extraction yield determinations, delignification rates, and crystallinity indices, were conducted in independent triplicates (n=3). Experimental values are expressed as mean ± standard deviation (SD). Statistical significance was determined via one-way analysis of variance (ANOVA), followed by Tukey’s Honestly Significant Difference (HSD) post hoc test utilizing OriginPro software. Differences were considered statistically significant at a threshold of p<0.05. Statistically distinct groups are denoted in data tables using lowercase alphabetical superscripts (^a–f^).

## 3. Results and Discussion

### 3.1. Chemical Composition of Raw Defatted SCGs

The chemical analysis of raw SCGs (Table 2) reveals a complex lignocellulosic matrix dominated by non-cellulosic constituents. The biomass contains 38% hemicellulose and 30.4% acid-insoluble lignin, while the cellulose fraction remains relatively low at 15.3%. This composition confirms the high recalcitrance of SCGs, where cellulose fibers are deeply embedded within a cross-linked matrix of hemicellulose and lignin. The high hemicellulose content reflects an abundance of amorphous polysaccharides, while the elevated lignin fraction contributes to the structural rigidity that limits cellulose accessibility. These results justify the application of synergistic alkali–urea–ultrasound pretreatments to selectively fractionate the biomass and enrich the cellulose yield. The presence of 1.9% ash is consistent with mineral residues commonly reported for agro-industrial waste and is not expected to significantly affect cellulose extraction.

The initial moisture content of the as-received wet SCGs was high (62.9%), which is characteristic of spent coffee grounds, therefore emphasizing the need to first dry these out before processing. Overall, the low initial cellulose content, combined with high lignin and hemicellulose fractions, justifies the application of synergistic alkali–urea–ultrasound pretreatments to selectively remove non-cellulosic components and enrich the cellulose fraction. These values are in good agreement with previously reported compositions of SCGs, confirming the representativeness of the raw materials used in this study.

Weight loss and extraction yield

The total weight loss was calculated by subtracting the dry mass of the SCGs before defatting (20 g) from the amount of extracted cellulose at the end of the extraction and bleaching process. This is cumulative of the process to remove non-cellulosic substances, such as lignin, hemicellulose, and extractives, instead of the changes in intermediate masses during specific processing stages, as shown in Table 3.

The relationship between the extraction yield and cellulose purity was quantitatively assessed using a mass-balance approach, in which the non-yield portion is attributed to the successful removal of hemicellulose (38%) and lignin (30.4%), thereby resolving the apparent contradiction between sample A and the synergistic treatments. Sample A (alkali-only) exhibited the highest recovery (32.3% ± 1.2). However, the mass-balance analysis indicated that this high yield is an artifact of incomplete delignification, with approximately 15–18% of the mass consisting of residual lignin and hemicellulose, as corroborated by the persistent FTIR signals at $1730\;cm^{-1}$ and 1510 cm^−1^. Sample C exhibited the lowest yield (15.0%), which is in line with the SEM findings of massive fragmentation and erosion, indicating that the cellulose was too disruptive to remain insoluble or be degraded. In contrast, sample F (5% NaOH + urea + ultrasound) demonstrated a significant reduction in yield to 26.0% ± 0.8, which corresponds to a delignification efficiency exceeding 85%. This demonstrates that the “lost” mass in synergistic treatments primarily represents the selective removal of the amorphous lignocellulosic matrix rather than the degradation of the cellulose backbone. The inverse correlation between yield and purity is thus statistically supported, as non-cellulosic components are removed through acoustic cavitation and urea-mediated swelling while the purity of the remaining cellulose fraction increases, as evidenced by the 12.5% increase in the crystallinity index (CrI) from sample A to sample F. The yield data indicates a critical trade-off, as while aggressive treatments enhance purity, they often lead to the degradation of cellulose chains and subsequent yield loss. Based on these findings, the low-alkali synergistic system (sample F) is considered optimal, as it balances satisfactory recovery with superior structural, thermal, and crystalline characteristics. The results of the study are also comparable to those reported for cellulose extracted using alkali and ultrasound-assisted techniques from lignocellulosic residues [10,12,24]. Reduced yields with subsequent increases in delignification have been observed in agricultural wastes, including coffee husk, rice straw, and sugarcane bagasse, with reductions of 15–30% depending on treatment severity [25,26,27]. The research has also indicated that alkali pretreatments with ultrasound increase the purity of cellulose at the cost of yield since they cause more fractional solubilization of amorphous fractions [10,22,28], which correlates appropriately with the trends in this case.

A detailed analysis is required to clarify the selection of sample F as the optimal process, considering that sample C achieved the highest absolute lignin removal (92.1% ± 1.1%) and sample B exhibited the highest apparent crystallinity index (64.5% ± 2.3%). Although sample C maximizes matrix purification, its high chemical severity (10% NaOH) caused extensive glycosidic chain cleavage and cellulose degradation, resulting in a low recovery yield of 15.0% ± 0.7%. Conversely, sample F (5% NaOH + 3% urea + ultrasound) balanced the process yield with chemical efficiency. By cutting the alkali requirement in half to 5% NaOH, it minimizes downstream chemical consumption, waste generation, and neutralization costs. Supported by the mechanical action of acoustic cavitation and urea-driven structural swelling, sample F yields a highly purified cellulose fraction (92.1% ± 1.1% purity) at a much higher yield of 26.0% ± 0.8%. This balanced outcome supports the selection of sample F as the optimal choice for localized, eco-friendly SME production.

### 3.2. Chemical Functionality (FTIR Analysis)

**Figure 1:**
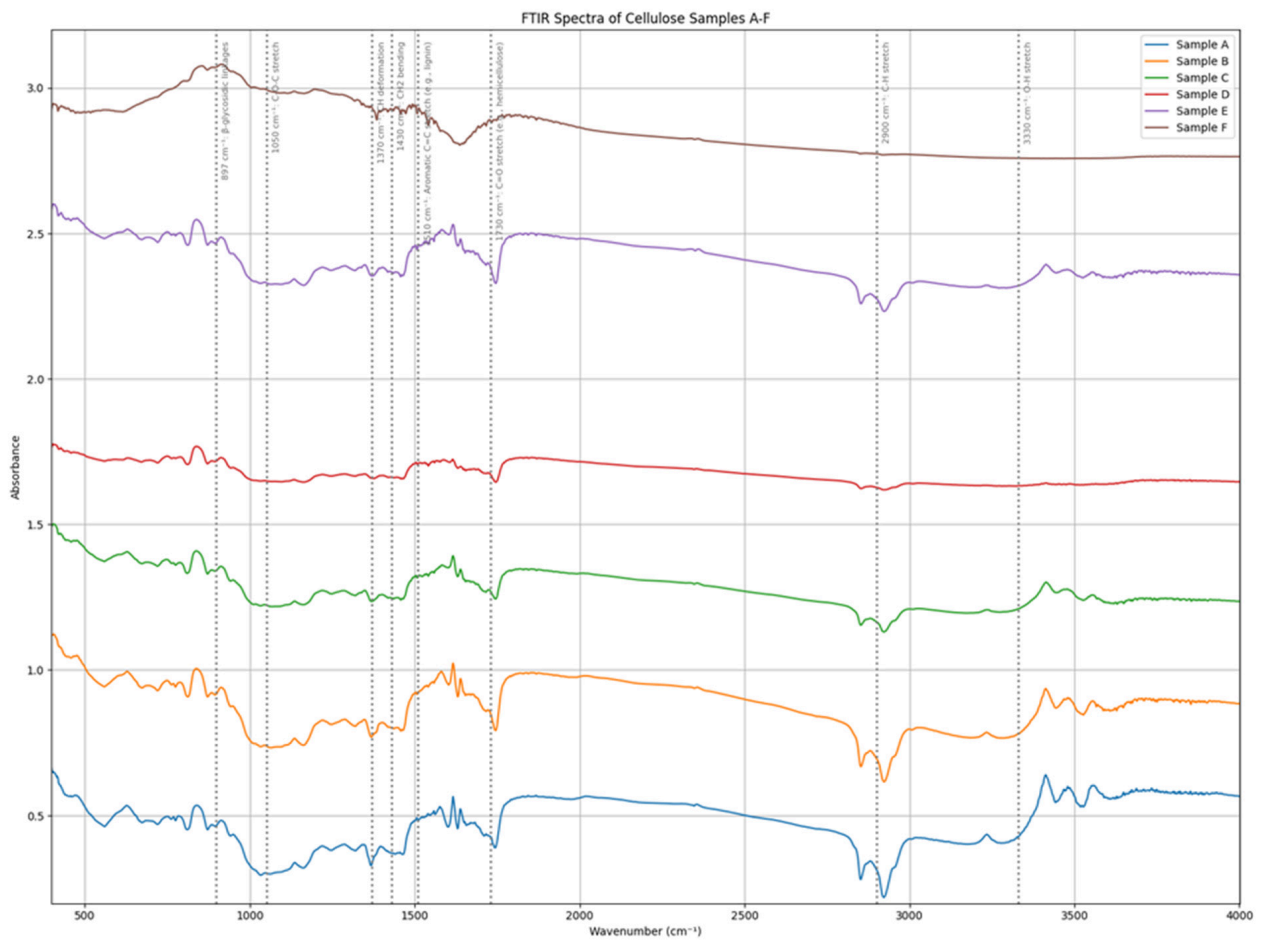
Illustration of the FTIR spectra of samples A–F, with distinct points at appx. 1730 cm^−1^ and 3330 cm^−1^.

The effectiveness of delignification and hemicellulose extraction was verified using FTIR spectroscopy. The bands of interest in the spectra were the OH stretch, representing the OH group (~3330 cm^−1^); the C-H stretch of the aliphatic groups (~2900 cm^−1^); and the C–O–C bands of the β-(1→4)-glycosidic bond (~1030 cm^−1^), all of which are cellulose structures. The loss of lignin and hemicellulose was confirmed by the disappearance or significant attenuation of aromatic skeletal vibrations (1510 cm^−1^, C=C) and carbonyl types of stretching at 1730 cm^−1^ (C=O of acetyl and uronic esters), which are hemicellulosic ester types and lignin derivatives. The ratio of the intensity of these peaks compared to that of the cellulose fingerprint region was used as a qualitative measure of the purification efficiency, as shown in Figure 1.

The alkali-only-treated SCGs in sample A had a clear reduction in the intensity of characteristic peaks at wavelengths of 1730 cm^−1^ (C=O stretching of acetyl and uronic ester groups) and 1510 cm^−1^ (C=C aromatic skeletal vibrations of lignin), reflecting the saponification of ester linkages and partial removal of hemicellulose. However, lignin-related bands were still detectable, suggesting that the alkali treatment alone did not fully disrupt the lignin–carbohydrate complex within the spent coffee matrix. These characteristics verify the heterogeneity of the untreated material chemically.

But with the addition of urea, further reductions in lignin-related absorptions of typical peaks at wavelengths of 1730 cm^−1^ (C=O stretching of acetyl and uronic ester groups) and 1510 cm^−1^ (C=C aromatic skeletal vibrations of lignin) occurred. This effect can be explained by the fact that urea has the potential to disrupt intermolecular hydrogen bonding, which encourages the swelling of fibers and increases the availability of reagents. Still, the existence of weak aromatic signals suggests that the chemical activity was constrained by the tight structure of the biomass.

The urea–ultrasound–NaOH mixed method, on the other hand, showed a strong attenuation and, in certain instances, complete disappearance of the bands at 1730 cm^−1^ and 1510 cm^−1^. This proves that it was more effective at eliminating hemicellulose and lignin. This was enhanced by ultrasonic cavitation, which physically breaks the cell wall structure and permits the alkali–urea system to penetrate into the lignin-rich areas. The treatment samples contained these in far less quantities and had no such properties in sample F, such as the successful cleavage of ester links and lignin structure degradation [29]. Separate cellulose absorption bands were preserved and further enhanced with treatment, as indicated by the broadening of O-H at 3330 cm^−1^, C-H at 2900 cm^−1^, and C-O-C pyranose ring vibration at 1030 cm^−1^. The 897 cm^−1^ β-(1→4)-glycosidic linkage peak also increased, which indicates the increase in the order and purity of the cellulose fractions [7]. These findings confirm the theory that the application of alkali–urea treatment, with the assistance of ultrasound, could promote the lignin and hemicellulose removability without influencing the cellulose integrity [30].

The FTIR analysis revealed that, while alkali treatment triggers the elimination of amorphous particles, the combined treatment of ultrasound and urea has the beneficial effect of significantly increasing the purification efficiency without cellulose chemical destruction. The FTIR results indicated that the alkali, urea, and ultrasound synergy was effective in the disruption of the lignin–carbohydrate complex, and this yielded cellulose containing few impurities. This equilibrium of successful delignification and structural conservation favors the appropriateness of the mixed treatment towards the production of cellulose with a regulated purity and stability.

### 3.3. Crystalline Structure (XRD)

**Figure 2:**
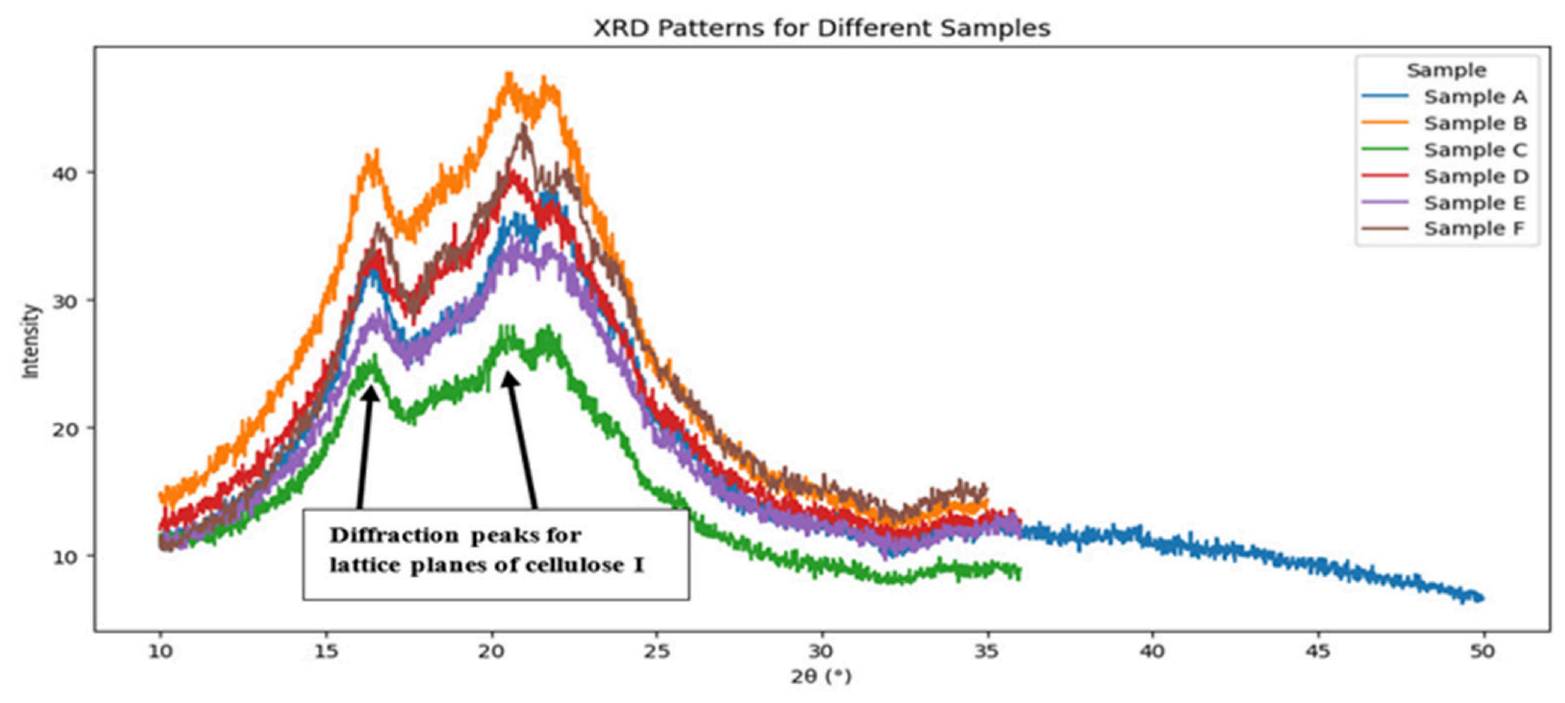
Illustration of the XRD patterns of samples A, B, C, D, E, and F, showing the expected peaks at about 2θ ≈ 16° (101) and 22° (002) corresponding to cellulose I.

To establish the crystalline characteristics of the extracted cellulose, X-ray diffraction (XRD) was performed. All the samples possessed the characteristic peaks (Figure 2) of cellulose I, with 2θ ≈ 16° (101) and 22° (002) as the crystalline planes of native cellulose [31]. Also, the absence peaks at either ~12° or ~20° confirmed there was no conversion to cellulose II, even after alkali–urea–ultrasound treatment. These characteristics ascertain that the extraction and pretreatment procedures had no effect on the crystalline polymorph of cellulose [32]. The samples that were subjected to ultrasound were found to have significantly sharper and stronger peaks than the samples extracted without ultrasound, a fact that implies an increase in structural order.

The Segal method was used to determine the crystallinity index (CrI). As the CrI rose, this showed higher removal of amorphous elements, like lignin and hemicellulose [33]. The same level of crystallinity has been observed to be improved by ultrasound-assisted cellulose extraction of other lignocellulosic sources [34].

While FTIR analysis confirmed the efficient removal of lignin and hemicellulose and the preservation of the cellulose backbone, XRD provided complementary information on the long-range crystalline organization of the cellulose chains. Crystallinity indices calculated using the Segal method ranged from 56.2% to 64.5% (Table 4), indicating partial enrichment of the crystalline cellulose fraction following pretreatments. These results suggest a synergistic effect between urea and ultrasound, primarily on chemical purification, where urea promotes fibril swelling and hydrogen-bond disruption, and ultrasound enhances mass transfer and delignification efficiency [24,33]. Nevertheless, the differences in crystallinity of the treated samples indicate variations in the microfibrillar packing, but not in chemical purity as such. Specifically, samples with a large portion of impurity removed by FTIR (e.g., sample C) had moderately decreased CrI values, which can be explained by the existence of ultrasound-induced dislocation of crystalline domains and a partial loss of long-range order. On the other hand, the increased values of CrI of some of the samples (e.g., sample B) could be due to the selective loss of amorphous regions rather than better chemical purification. In general, the CrI values were moderate (that is, 56–59%), which means that amorphous components were partially excluded, and cellulose fibrils were not entirely rearranged or recrystallized. This is a synergistic effect between urea (fibril swelling and disruption of hydrogen bonds) [24] and ultrasound (increasing the mass transfer and efficacy of delignification) [33].

These results are in line with past reports that have indicated that ultrasound-aided alkali–urea systems can purify cellulose and, at the same time, control the level of crystalline order through mechanical disintegration of the lignocellulosic skeleton [11,16,34,35,36]. The resulting cellulose, which maintains its cellulose I structure, moderate crystallinity, and high chemical purity, is a promising raw material to be used in higher-value processes, such as composite reinforcement and nanocellulose. When combined, FTIR and XRD measurements indicate that the alkali–urea–ultrasound treatment is suitable to optimize cellulose purity and, at the same time, control the crystalline structure of cellulose.

The lower crystallinity index (CrI) of sample F (56.2% ± 2.4%) relative to sample B (64.5% ± 2.3%) highlights a key structural trade-off between treatment conditions. In sample B (10% NaOH + ultrasound), the higher alkali concentration dissolves the amorphous lignin and hemicellulose matrices without penetrating the crystalline cores, leading to a high apparent crystallinity index. In sample F, however, the inclusion of 3% urea introduces a strong hydrogen-bonding agent that disrupts the internal hydrogen-bonding networks, stabilizing the native cellulose microfibrils. This mechanism promotes significant fiber swelling and induces localized structural amorphization. As a result, sample F displays a lower long-range crystalline order in XRD analysis despite having high chemical purity, as shown by FTIR.

### 3.4. Thermal Stability (TGA and DTG)

**Figure 3:**
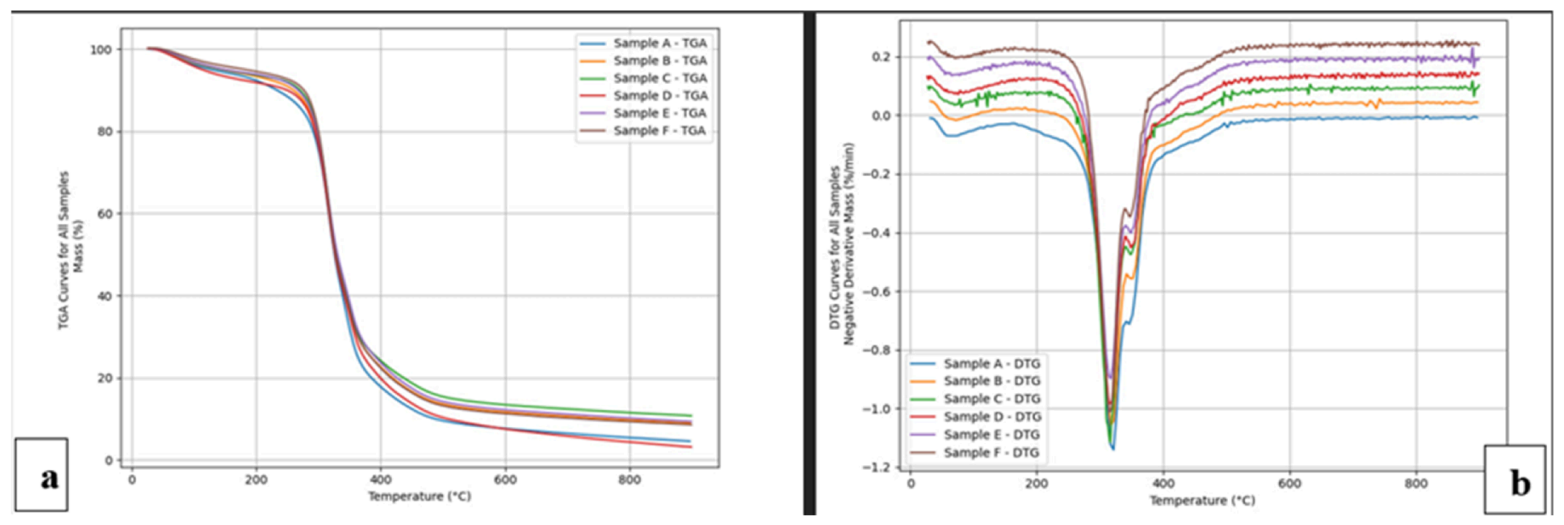
(**a**,**b**) TGA and DTG thermograms of cellulose samples (A, B, C, D, E, and F), showing distinct two-step degradation and single-peak DTG behavior.

Thermal degradation kinetics provide invaluable data about the stability during processing and the behavior of the extracted cellulose in terms of its decomposition. The typical thermograms had three main weight loss peaks: (i) moisture evaporation less than 120 °C, indicating the physically adsorbed water; (ii) major decomposition between 250 and 370 °C, indicating the chain depolymerization and cleavage of glycosidic bonds of cellulose; and (iii) char formation at or above 400 °C, indicating the formation of carbonaceous residues. The onset temperature (T_onset_) and the maximum degradation temperature (T_max_) were determined by using the derivative thermogravimetric (DTG) curves [8]. T_onset_ and T_max_ of the refined cellulose were higher than those of raw SCGs, which showed the removal of thermally unstable hemicellulose and lignin constituents. This rise in thermal resistance was confirmation of the structural integrity and purity of the achieved cellulose [13,23]. The thermogravimetric (TGA) and derivative thermogravimetric (DTG) analyses (Figure 3) indicated that the ultrasound-assisted alkali–urea process is effective in maintaining the stability of cellulose by showing a two-step degradation characteristic of high-quality cellulose produced through the green pretreatment routes. The initial phase below 100 °C is the moisture desorption phase, while the major decomposition phases are 250 °C and 370 °C, which are the glycosidic bond scission and the volatile low-molecular-weight fragment stages. Table 4 summarizes the peak decomposition temperatures (T_max_) and overall mass losses, and shows that all the T_max_ values are concentrated within a narrow range of 316–322 °C, indicating that the extracted samples of cellulose are thermally homogeneous. In contrast to sample D (10% NaOH + urea + ultrasound), which showed the greatest total mass loss (97.0%), indicating a high-purity cellulose fraction with only a small amount of residual char, sample A had the highest Tmax (321.6 °C), which was probably caused by the presence of residual lignin promoting char formation and slowing down thermal decomposition [37].

For further clarity, kinetic modeling was performed using the Kissinger method to estimate the apparent activation energy (Ea). It is explicitly assumed that cellulose degradation follows first-order reaction kinetics, where the rate of weight loss is proportional to the remaining mass of the reactant; hence, the expression below was used:

Ln (β/Tm2) = −(Ea/RTm] + Ln (AR/Ea)

where β = the heating rate (K/min); Tm = the peak decomposition temperature (K); R = the universal gas constant (8.314 J/mol·K); A = the pre-exponential factor (s^−1^); and Ea = the apparent activation energy (kJ/mol).

The Ea values were estimated using experimental Tmax data with a heating rate of 10 °C/min, and these values were then compared to the literature data on the decomposition of lignocellulosic cellulose [32].

Kinetic and Structural Correlation:

From Table 4, the activation energies (Eas) of 168.5 to 185.2 kJ·mol^−1^ were consistent with the literature’s values of thermally purified cellulose (160–190 kJ·mol^−1^) [30]. The thermogravimetric analysis showed that the main step in the degradation process was between 316 and 322 °C, which is associated with the depolymerization of cellulose. Tmax and activation energy differences between the samples indicate the differences in chemical composition and crystalline structure.

Samples that still had lignin traces had greater activation energies because of the thermally stable aromatic forms of lignin; this was found in sample A, which had a slightly higher Ea, suggesting that this resistance was related to residual lignin. The thermal stability of these samples is proven by the absence of hemicellulose and amorphous lignin fractions, which degrade at lower temperatures [34]. The Ea increase in the ultrasound-assisted and urea-modified treatments is evidence of the better molecular arrangement and thermal solidification of the extracted cellulose, which is consistent with the increased crystallinity index (CrI) of the XRD analysis. Sample F had the perfect proportion of activation energy and purity, and thus the applicability of the alkali–urea–ultrasound system to purify cellulose with favorable thermal stability and activation energy barriers. This system may be potentially used in thermo–mechanical composites and biopolymers to improve processing performance, according to these calculated kinetics [30,38,39].

It is important to note that sample C, which exhibited the best lignin and hemicellulose removal by FTIR, had the lowest Ea. The cause of this behavior was the disruption of the bonding and the decreased crystal coherence because of ultrasounds, as supported by XRD. Sample E, on the other hand, displayed a balanced thermal stability, which was a result of a good trade-off in chemical purity and structural order. Intermediate crystallinity and chemical purity yielded balanced thermal behavior of the samples, meaning that an excessive elimination of amorphous space or excessive disruption of crystalline space can adversely affect thermal stability. These findings indicate the strong interdependence between chemical purification, supramolecular structure, and thermal performance. While this method is robust for assessing the thermal solidification of refined cellulose, its limitation is that lignocellulosic biomass is a multi-component system, and the Kissinger approach may simplify the complex, overlapping degradation pathways of residual hemicellulose or lignin traces. Nevertheless, the Ea values obtained (168.5 to 185.2 kJ·mol^−1^) align with the literature’s standards for purified cellulose, confirming the structural integrity of extracted fibers.

### 3.5. Morphological Analysis (SEM)

**Figure 4:**
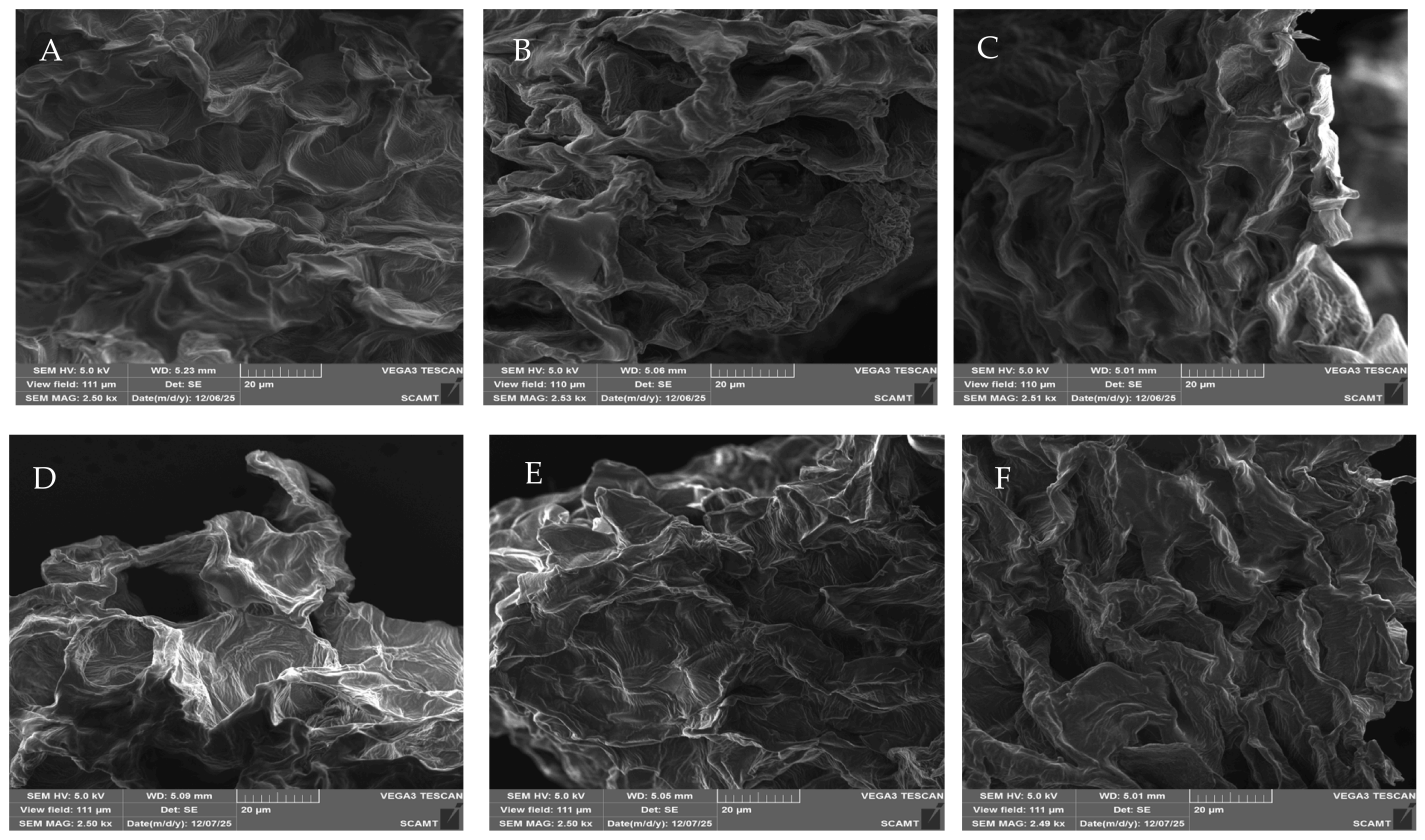
SEM micrographs illustrate morphological transformation from compact lignin-rich surfaces (samples A, B and C) to fibrillated cellulose networks (samples D, E, and F).

SEM imaging (Figure 4) visually corroborates the structural transitions identified via FTIR and XRD. The raw SCGs (sample A) exhibited a dense, compact, and heterogeneous morphology, characteristic of a matrix where lignin and hemicellulose act as binders for polysaccharide components [14]. Following synergistic chemical and ultrasonic treatment, significant morphological degradation was observed. The surfaces showed progressive roughening, fragmentation, and the emergence of loosely packed granular structures. This transition indicates the successful disruption of lignin–carbohydrate complexes and the fibrillation of cellulose microfibril bundles, which is facilitated by ultrasonic cavitation enhancing reagent penetration and mass transfer.

Sample A (treated with alkali alone) exhibited a thick, compact surface with a smooth fiber bundle—a feature of a strongly formed lignin-coated surface—and limited accessibility to the fibers. The lignin and lipid coating resulted in the high density and compact surface of the untreated SCGs. Great morphological changes were noticed after treatments using chemicals and ultrasound, including the progressive roughening of surfaces, fragmentation, and appearance of loosely packed platelet-like and granular structures on treated samples, instead of the retention of fibrous networks. This morphology is an indicator of the lignin–carbohydrate complexes’ disruption and the slight disintegration of cellulose microfibril bundles under the influence of alkaline and ultrasonic conditions, which enhanced the transfer of mass through extraction and bleaching [12]. The samples treated were fibrillated and coarse in nature, with clear lignin removal and an exposed cellulose fibril network [14].

Moderately treated samples (samples B, D, and E) had more homogeneous particle distributions and a higher surface porosity or compactness, as is often the case in other comparable studies [40]. These characteristics report good lignin and hemicellulose removal and improved exposure of cellulose domains, which are consistent with FTIR data of lignin and hemicellulose removal and XRD data of increased apparent crystallinity. Sample C, on the contrary, was highly fragmented and had surface erosion, which was aligned with the disruption of hydrogen bonding and a decrease in microfibrillar coherence induced by ultrasound, as indicated by a moderate index of crystallinity and a lower energy of activation following TGA.

Sample F (urea and ultrasound pretreatments), on the other hand, has reduced albeit highly aggregated structures, indicating the overtreatment and hornification effect, which are associated with a lesser intensity of cellulose fingerprints in FTIR and with a lesser crystallinity index. The collapse of microbubbles causing microstructural erosion in ultrasonic cavitation was what physically disintegrated the lignin–carbohydrate complex and augmented surface roughness and porosity [24]. Nevertheless, too much acoustic cavitation and chemical swelling facilitated the aggregation and collapse of the fibrillated structures, as opposed to the formation of a loosely held network of microfibrils. The description of such surface features justifies the lack of enhanced XRD crystallinity, showing that even amorphous matrix extraction was maintained; and yet, the ordered cellulose domains were partially broken under severe treatment conditions [34].

The morphological variations observed in the SEM micrographs (Figure 4) visually support the underlying chemical and structural properties measured via FTIR, XRD, and TGA. The surface erosion, roughening, and emergence of loosely packed granular matrices in samples D, E, and F cannot be interpreted as isolated surface effects. These texturing changes directly correlate with the complete attenuation of the hemicellulose carbonyl band ($1730{\;\mathrm{cm}}^{-1}$) and aromatic lignin vibrations ($1510{\;\mathrm{cm}}^{-1}$) observed in the FTIR spectra, confirming that the physical changes are driven by the removal of the amorphous binder matrix. Similarly, the highly fragmented and eroded surfaces seen in sample C explain its low thermal activation energy ($168.5\;\mathrm{kJ}\cdot {\mathrm{mol}}^{-1}$) and lower crystal coherence in XRD. This directly confirms that severe ultrasonic cavitation breaks down long-range microfibrillar packing, matching the structural trends identified across the other analytical techniques.

## 4. Conclusions

The research predetermined a sustainable and effective approach for the extraction of cellulose in spent coffee grounds (SCGs) using a synergistic approach involving alkali pretreatment, urea modification, and ultrasonic irradiation. Extensive characterization using FTIR, XRD, TGA/DTG, and SEM indicated that although traditional alkali treatment eliminated a portion of non-cellulosic substances, it was not capable of completely destabilizing the compact lignocellulosic structure of SCGs. Conversely, urea and ultrasound addition greatly improved the process of delignification and hemicellulose extraction, as demonstrated by the faded FTIR bands, the low amorphous quantities in XRD, and the altered thermal degradation pattern. XRD analysis established the maintenance of the cellulose I polymorph in all treatments, and crystallinity indices were between 56.2% and 64.5%, which means some amorphous areas were removed but cellulose fibers were not fully rearranged. Crystallinity differences were associated with differences in microfibrillar packing, which was caused by the severity of the treatment and not by chemical purity. The thermal analysis revealed a prevailing degradation step between 316 °C and 322 °C, with activation energies comparable to those of thermally purified cellulose. The thermal stability of samples with balanced crystallinity and chemical purity was positive, which indicated the significance of the intensity of treatment control. The spectroscopic and thermal findings were corroborated by direct morphological evidence in SEM observations showing a progressive degradation of the dense lignocellulosic structure, massive defibrillation, roughing of surfaces, and augmentation of porosity after alkali–urea–ultrasound treatment. There were no clear fibers signifying fragmentation and fibrillation nor fiber isolation, as shown by this absence of clear fibers. This is in line with the increased accessibility of cellulose domains. Interestingly, the ultrasound system that was used (low-alkali synergistic system of 5% NaOH + urea + ultrasound) yielded cellulose with crystallinity and thermal stability equal to that of treatments using higher chemical concentrations, showing that ultrasound allows for the reduction in chemical loading while the material performance remains the same. Moreover, ultrasonic irradiation significantly reduced the time of extraction by increasing the velocity of mass transfer and decomposing lignin–carbohydrate complexes faster, thereby increasing the sustainability and efficiency of the process.

In conclusion, this study provides a rigorous quantitative framework for the valorization of SCGs. By reconciling the trade-off between yield and purity, we have demonstrated that the alkali–urea–ultrasound system (specifically the low-alkali sample F) is superior for producing high-purity cellulose I. While traditional methods (sample A) appear more efficient in terms of raw mass recovery, our characterization proves that this mass largely comprises impurities. The synergistic approach not only achieves higher delignification but does so with greater energy efficiency and reduced chemical loading, facilitating the development of sustainable, high-purity biopolymers for the circular bioeconomy.

## Figures and Tables

**Table 1:** Pretreatment key depicting how samples A, B, C, D, E, and F were treated during the experiment procedure.

Treatment	NaOH (%)	Urea (%)	Ultrasound	Notes
[A]	10	0	No	Conventional alkali
[B]	10	0	Yes	Alkali + ultrasound
[C]	10	3	No	Alkali + urea
[D]	10	3	Yes	Alkali + urea + ultrasound
[E]	5	3	No	Low-alkali + urea
[F]	5	3	Yes	Low-alkali + urea + ultrasound

**Table 2:** The chemical composition of raw SCGs, shown in percentages of component compositions.

Component (%)	Raw SCGs
Cellulose	15.3±1.8
Hemicellulose	38±2.5
Lignin (acid-insoluble)	30.4±1.6
Ash	1.9±0.2
Moisture	62.9±4.0

Values are expressed as means ± standard deviations (SD) based on triplicate measurements for each sample.

**Table 3:** Statistical comparison of cellulose yield, delignification, and purity.

Sample	Final Weight (g)	Weight Loss (g)	Cellulose Yield (%)	Lignin Removal (%)	Cellulose Purity (%)
A	6.457	13.543	$32.3\pm 1.2^{\;a}$	$45.2\pm {2.5\;}^{f}$	$52.4\pm 2.1^{\;e}$
B	3.793	16.207	$18.9\pm 0.9^{\;d}$	$78.4\pm 1.8^{\;d}$	$85.6\pm 1.4^{\;c}$
C	3.008	16.992	$15.0\pm 0.7^{\;e}$	$92.1\pm 1.1^{\;a}$	$94.8\pm {0.9\;}^{a}$
D	3.306	16.694	$16.5\pm 1.1^{\;\mathrm{de}}$	$90.5\pm 1.4^{\;\mathrm{ab}}$	$93.2\pm 1.2^{\;\mathrm{ab}}$
E	3.925	16.075	$19.6\pm {0.8\;}^{\mathrm{cd}}$	$72.3\pm 2.0^{\;e}$	$81.5\pm 1.7^{\;d}$
F	5.201	14.799	$26.0\pm 0.8^{\;b}$	$88.5\pm 1.5^{\;b}$	$92.1\pm 1.1^{\;b}$

Values are expressed as means ± standard deviations (SD) based on triplicate measurements. In contrast to distinct letters within the same column denoting a significant disparity between treatments, identical letters signify no significant variances between treatments (*p* < 0.05).

**Table 4:** Thermogravimetric parameters and crystallinity indices of extracted cellulose samples.

Sample	Tmax (°C) ± 3.0	Tmax (K)	Total Mass Loss (%)	Ea (kJ⋅mol^−1^) ± 8.0	Crystallinity Index CrI (%)
A	321.6	594.6	95.47	$185.2^{\;b}$	$60.5\pm 2.1^{\;b}$
B	317.1	590.1	91.12	$173.4^{\;cd}$	$64.5\pm 2.3^{\;a}$
C	316.2	589.2	89.38	$168.5^{\;d}$	$59.2\pm 2.0^{\;\mathrm{bc}}$
D	316.0	589.0	97.00	$178.7^{\;bc}$	$61.1\pm {2.2\;}^{b}$
E	317.1	590.1	90.91	$170.2^{\;d}$	$60.5\pm 2.1^{\;b}$
F	316.3	589.3	91.72	$176.9^{\;c}$	$56.2\pm 2.4^{\;c}$

Values are expressed as means ± standard deviations (SD) based on triplicate measurements. In contrast to distinct letters within the same column denoting a significant disparity between treatments, identical letters signify no significant variances between treatments (*p* < 0.05).

## Data Availability

The original contributions presented in this study are included in the article. Further inquiries can be directed to the corresponding authors.
